# Physical Functioning, Physical Activity, and Variability in Gait Performance during the Six-Minute Walk Test

**DOI:** 10.3390/s24144656

**Published:** 2024-07-18

**Authors:** Julie Rekant, Heidi Ortmeyer, Jamie Giffuni, Ben Friedman, Odessa Addison

**Affiliations:** Baltimore VA Medical Center, Baltimore Department of Physical Therapy and Rehabilitation Sciences, University of Maryland, Baltimore, MD 21201, USA; hortmeye@som.umaryland.edu (H.O.); jamie.giffuni@va.gov (J.G.); ben.friedman@som.umaryland.edu (B.F.); oaddison@som.umaryland.edu (O.A.)

**Keywords:** fall risk, six-minute walk test, gait performance, four square step test, wearable sensors

## Abstract

Instrumenting the six-minute walk test (6MWT) adds information about gait quality and insight into fall risk. Being physically active and preserving multi-directional stepping abilities are also important for fall risk reduction. This analysis investigated the relationship of gait quality during the 6MWT with physical functioning and physical activity. Twenty-one veterans (62.2 ± 6.4 years) completed the four square step test (FSST) multi-directional stepping assessment, a gait speed assessment, health questionnaires, and the accelerometer-instrumented 6MWT. An activity monitor worn at home captured free-living physical activity. Gait measures were not significantly different between minutes of the 6MWT. However, participants with greater increases in stride time (ρ = −0.594, *p* < 0.01) and stance time (ρ = −0.679, *p* < 0.01) during the 6MWT reported lower physical functioning. Neither physical activity nor sedentary time were related to 6MWT gait quality. Participants exploring a larger range in stride time variability (ρ = 0.614, *p* < 0.01) and stance time variability (ρ = 0.498, *p* < 0.05) during the 6MWT required more time to complete the FSST. Participants needing at least 15 s to complete the FSST meaningfully differed from those completing the FSST more quickly on all gait measures studied. Instrumenting the 6MWT helps detect ranges of gait performance and provides insight into functional limitations missed with uninstrumented administration. Established FSST cut points identify aging adults with poorer gait quality.

## 1. Introduction

Changes in gait performance are common in aging adults and can be used as markers of physical decline [1]. However, standard clinical assessment of gait typically provides gross measures of mobility, like walking speed and distance covered during a set walking period, and does not provide insight into the quality of how people are performing these tasks. Gait performance measures, like spatiotemporal characteristics of gait and gait variability, unveil mechanisms of motor control and dynamic balance contributing to gait quality and fall risk [2,3]. Instrumented walking assessments with inertial sensors have been shown to accurately and reliably capture step-to-step alterations in healthy and diseased populations [4,5] and are recommended to improve clinical evaluation and prognostics [6]. In particular, instrumenting the six-minute walk test (6MWT) with wearable inertial sensors can “increase the meaningfulness of the 6MWT” by providing greater, more detailed information on step-to-step gait performance [4]. Understanding what is happening during each step of the 6MWT gives information on control mechanisms behind dynamic balance during walking and allows clinicians to identify trends in how gait performance declines as individuals fatigue.

In adults with multiple sclerosis, an instrumented 6MWT distinguishes distinct trajectories in gait performance measures between those with mild and moderate disability [7]. An instrumented 6MWT can also be used to identify if particular times during the prolonged walking test have stronger diagnostic implications than other minutes of the test to better direct clinical decision-making. For example, Grimpampi et al. found that comparing stride duration and vertical trunk accelerations from the first to the last minute of the 6MWT revealed significant decreases in gait performance among older adults [8], which may indicate fatigue or balance declines. This has also been shown with physiological measures. SpO_2_ during the third minute of the 6MWT has shown the strongest correlation with lung diffusing capacity in adults with interstitial lung disease [9], indicating that performance during the middle minutes of the 6MWT may provide meaningful insight into physiological functioning. However, an instrumented assessment of the 6MWT has not yet been performed in conjunction with functional testing to establish these associations in healthy aging adults.

Taking more time to complete steps during the gait, spending more time in the stance phase of each gait cycle, and having higher step-to-step variability are indicative of poor dynamic balance, decreased walking skill, and increased fall risk [10,11]. Engaging in more moderate-vigorous physical activity (MVPA) and less sedentary activity has shown protective effects in aging adults against physical and physiological decline [12,13]. The four square step test (FSST), a simple clinical assessment of dynamic balance and direction-changing abilities [14], has also been shown to detect fall risk among older adults [14,15]. Among adults at high risk of disability development, it can be difficult to distinguish functional levels. Maintaining physical activity levels and multidirectional stepping abilities means older adults are less likely to experience falls and develop disability [14,15,16], indicating that MVPA and the FSST might be appropriate group classifiers, providing clinical value in conjunction with the instrumented 6MWT. However, there is a paucity of evidence combining the instrumented 6MWT with measures of physical functioning and physical activity to disentangle this.

The purpose of this study was to evaluate how measures of gait performance captured during an instrumented 6MWT relate to physical performance, health measures, and observed physical activity level. Additionally, the value of using functional tests as group classifiers to detect distinct profiles of gait performance during the 6MWT was explored.

## 2. Materials and Methods

Participants were recruited through flyers and referrals from their medical provider as veterans interested in potential health and wellness programming at the Baltimore Geriatric Research Education and Clinical Center (GRECC). Veterans were eligible if they were 50 years of age or older, received care at the VA Maryland Health Care System, had VA primary care approval to begin a program of physical activity, were able to walk independently with or without an assistive device, were medically stable, and were without cognitive impairment, unstable angina, proliferate diabetic retinopathy, oxygen dependency, frank incontinence, open wounds, and/or active substance abuse. All study activities were approved by the University of Maryland Institutional Research Board (HP-97664) and the Veterans Affairs Research and Development Committee (1651218). The participants signed informed consent prior to participation in research study activities.

### 2.1. Functional Testing

**Figure 1:**
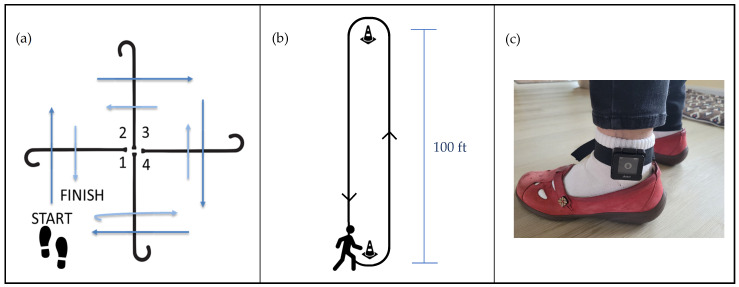
Functional testing setup demonstrating the (**a**) four square step test, (**b**) six-minute walk test, and (**c**) wearable sensor configuration for the six-minute walk test.

All physical function testing was performed by trained Baltimore VA Medical Center staff. Anthropometry was collected, then the participants completed the Short Form (36) Health Survey Physical Function Subscale (SF-36) [17] and PROMIS Global Health Scale (GHS) [18] self-reported quality-of-life questionnaires. The participants then completed functional assessments of balance, mobility, and endurance including the four square step test (FSST) [14], a test of usual gait speed over 10 m, and the six-minute walk test (6MWT) [19]. The FSST is an assessment of multidirectional stepping where participants are asked to step over canes arranged in a “plus” configuration, first navigating all four spaces in a clockwise direction, then immediately in a counter-clockwise direction as quickly as possible, while facing forward and avoiding hitting the canes (Figure 1a). FSST performance has been shown to discriminate fallers from non-fallers. For the 6MWT, participants are asked to walk as far as possible in six minutes around a 100 ft course (Figure 1b); participants were allowed to take standing rests during the six minutes, if needed, then continue walking. If participants requested to stop the test or sit before the end of six minutes, only distance traversed prior to this seated break was included. The participants were equipped with an ActiGraph GT9X Link monitor (ActiGraph, Pensacola, FL, USA) on their left ankle to collect stride-to-stride limb accelerations during the 6MWT (Figure 1c).

### 2.2. Physical Activity Assessment

The participants were provided the ActiGraph GT9X Link monitor (ActiGraph, Pensacola, FL, USA) and instructed to wear it on the wrist at home 24 h a day to capture regular physical activity. All ActiGraph data were collected at 100 Hz using ActiLife v6.13.4 software (ActiGraph, Pensacola, FL, USA).

Raw accelerometry was extracted from the ankle-worn ActiGraph worn during the 6MWT and was parsed into steps with a custom MATLAB code (Version R2022a, The MathWorks, Inc., Natick, MA, USA). Data were filtered with a 2nd-order 5 Hz low-pass Butterworth filter, and gait events were identified as peaks from vector magnitudes [20]. All gait events were visually verified by JR. Standing rest breaks during the 6MWT were excluded from the analysis. Stride time, stance time, stance percentage, swing time, and swing percentage were calculated in accordance with the previous literature [21]. The variability of each gait performance measure was quantified with the coefficient of variation (COV) ((standard deviation/mean) × 100).

Six measures of gait performance were selected to represent different domains of gait: pace (swing time variability), postural control (stance percentage), rhythm (stride time, stance time), and variability (stride time COV, stance time COV) [22]. Each measure was summarized to present within-minute mean performance, and changes in gait performance across the 6MWT time were calculated as the maximum minus the minimum of these within-minute mean values for each metric [23]. The minute when each maximum and minimum value was observed was also recorded.

ActiGraph data from the wrist-worn monitor during the at-home monitoring period were processed with the low-frequency extension [24] and screened for wear time [25]. Raw data from the initial 24-h wear period were included in the analysis. Time spent in each of the following periods was calculated for the 24-h wear period per Montoye cut points: sedentary, light, and MVPA [26]. Sleep periods were identified with the Cole–Kripke algorithm [27] and visually verified by BF. Time spent in sleep was subtracted from sedentary activity time. Activity data are presented as percentages of the 24-h wear period.

A one-way analysis of variance (ANOVA) was performed on all within-minute measures to identify whether a time effect of minutes during the 6MWT existed. Spearman correlations were performed to explore the relationships between clinical measures and measures of physical activity, and changes in gait performance. In order to best evaluate the effect of fall risk on gait performance, the participants were grouped based on their FSST times into fast (≤12 s), medium (12.01–14.99 s), and slow (≥15 s) groups [14,15,28]. To understand if the timing of follow-up, functional status at baseline (defined by FSST group), and/or the interaction of these factors drove changes in gait performance, a repeated measures ANOVA (RMANOVA) was performed with changes in gait performance as the outcome, the main effects of time (baseline vs. follow-up) and FSST group (fast vs. medium vs. slow), and an interaction effect of time × FSST group. Post hoc *t*-tests with Bonferroni corrections for multiple comparisons were performed.

## 3. Results

Twenty-one veterans referred to the GRECC health and wellness program were eligible for the study and consented to participate. The participants, on average, had obesity and were primarily male and Black or African American. Detailed participant demographics can be found in Table 1.

**Figure A1:**
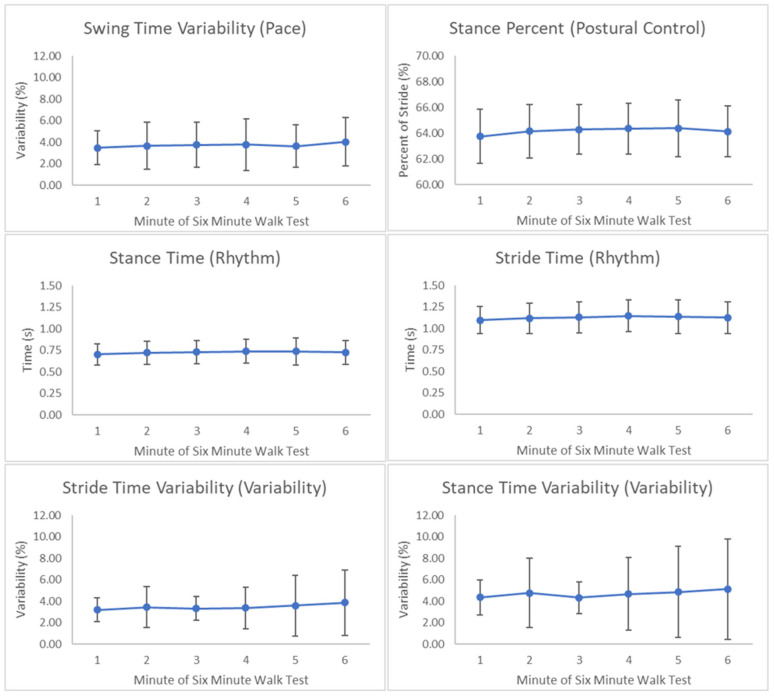
Change in gait performance measures across the six-minute walk test. No statistically significant effects of minutes were observed.

The participants reported below-average physical functioning on the SF-36 Physical Function subscale (59.05 ± 20.71 out of 100). This is lower than both the mean reported for the U.S. population (84.15 ± 23.28) [29] and for older adults (74.7 ± 19.4) [30]. The study participants’ mean GHS T-scores were 37.7 ± 10.0 on the Global Physical Health subscale and 41.3 ± 7.0 on the Global Mental Health subscale. These values are one standard deviation lower than the mean for similarly aged adults [31]. The participants completed the FSST in an average of 13.39 ± 4.96 s, had an average gait speed of 1.19 ± 0.41 m/s, and covered an average distance of 430.63 ± 111.56 m on the 6MWT. Three participants took standing rest breaks during the 6MWT (35.68 s, 79.95 s, 10.38 s). One participant’s standing rest break lasted the entire fourth minute of the 6MWT, so only 20 participants are included in the analysis for minute 4. A summary of within-minute gait performance measures can be found in Table 2 and visually in Figure A1. Gait performance measures did not significantly change across the minutes of the 6MWT.

The amount of change in gait performance measures observed during the 6MWT was significantly associated with having a slower gait speed, taking more time to complete the FSST, covering a shorter distance during the 6MWT, and having lower self-reported physical functioning (Table 3). Neither summary measures of global health nor physical activity were significantly related to 6MWT performance.

There was a wide spread in when maximum and minimum values in gait performance were observed (Table 4). The participants had the shortest stride and stance times in the first minutes of the 6MWT and demonstrated the longest stride and stance times during or after the third minute. The greatest amount of variability in swing time occurred later in the 6MWT; however, maximum and minimum swing time COV values were observed across all minutes of the 6MWT. Stance time COV maximums were rarely observed during the fourth minute of the test, and minimums were rarely observed in the third minute of the test. No consistent patterns were observed for when minimum and maximum values for stance percentage and stride time COV occurred.

**Figure A2:**
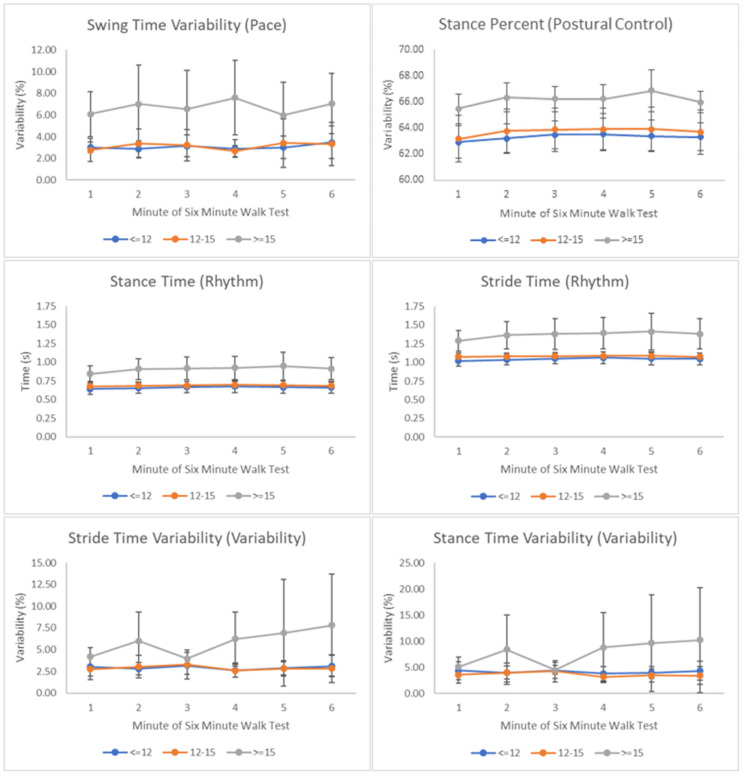
Gait parameters significantly differed for the slow FSST group when compared to the fast and medium groups.

An exploratory analysis was performed to investigate whether clinical cut points on the FSST could identify different trajectories in gait parameters during the 6MWT. A significant effect of FSST group, but not time, was observed for all gait parameters (Table 5, Figure A2). Post hoc testing identified the slow FSST group (taking ≥15 s to complete the test) as significantly different from both the fast (≤12 s) and medium (12–15 s) groups for all gait parameters assessed (Table 6).

## 4. Discussion

An instrumented 6MWT provides information on gait performance changes over the testing period. The present analysis explored the relationship between gait performance changes during the 6MWT and measures of mobility in aging veterans, presenting a novel exploration combining instrumented assessments to better understand physical functioning. The main finding in this study was that, while gait performance measures did not differ across the minutes of the 6MWT (Table 2), changes in gait performance during the test were meaningfully related to performance-based and self-reported measures of mobility and physical functioning (Table 3). A secondary analysis showed veterans who completed the FSST slowly (≥15 s) had meaningfully different gait performance during the 6MWT compared to those who completed the FSST in 12.01–14.99 s or faster than 12.01 s (Table 6), though the authors recognize conclusions from this finding may be limited due to the small sample size.

Aging veterans with larger changes in gait variability during the 6MWT also had slower self-selected gait speeds, covered less distance on the 6MWT, and required more time to perform the FSST. These findings are consistent with the previous literature which reports greater gait variability among those with slower gait speeds and impaired mobility [32,33]. Practically, this makes sense as walking with a slower gait speed over the 6MWT will also mean covering a shorter distance during the test. Lower self-reported physical functioning was associated with greater increases in stride time and stance time across the 6MWT. This was expected as spending more time in stance and taking longer to complete each stride are thought to be adaptations aimed at increasing stability in those with lower physical functioning, poor balance, and increased fall risk [34].

Contrary to expectations, changes in gait performance during the 6MWT were not related to physical activity level in this analysis. Previous work has found positive associations between 6MWT distance and physical activity level in healthy and diseased aging adults [35,36]. However, these analyses used self-reported physical activity level as opposed to the objectively measured physical activity reported in the present analysis. Though self-reported physical activity may be appropriate for larger epidemiological studies, it has been found to be inaccurate and unreliable for analyses at the individual level [37]. The present analysis objectively measured physical activity in the participants’ home environments; however, it is possible there was not a wide enough distribution in physical activity levels among the aging veterans included in the present analysis for an association to be observed. Further, it is possible the summary measures of physical activity level included in the analysis (daily percentage of sedentary and moderate–vigorous activity) did not capture domains of physical activity which would translate into gait performance during a prolonged walking task. Future work should explore the association of measures like activity types, length of active and sedentary bouts, and activity fragmentation [38] with gait performance changes during the 6MWT to better understand the association of physical activity quality with gait quality.

When maximum and minimum values of gait performance measures observed during the 6MWT varied widely (Table 4).As expected, there were trends towards temporal characteristics being at their quickest earlier in the 6MWT and becoming slower later in the test. Step time slowing during prolonged walking tasks is considered a measure of fatigability [39] and is an anticipated effect of performing the 6MWT. No clear patterns were identified for when extreme temporal characteristic variability values occurred during the 6MWT. Variability itself did not change meaningfully over the minutes of the 6MWT; however, individuals with larger changes in gait variability measures during the 6MWT also demonstrated poorer functional performance during clinical testing (Table 3). This indicates an individualized approach to assessment is needed to pinpoint when older adults are experiencing difficulty during prolonged activity and what the functional implications of this might be. Adding wearable sensors to standard clinical assessments is a low-cost, simple way to provide high-quality, individualized assessment outside of research environments and is recommended for future work. Clinicians can use this individualized information for targeted gait training for fall risk reduction.

The time taken to complete the FSST has been shown to discriminate multiple fallers (≥15 s) [19] from non-multiple fallers (12.01–14.99 s) [15] and non-fallers (≤12 s). Veterans who completed the FSST slowly (≥15 s) had greater gait variability and took slower steps than the medium (12–15 s) and fast (≤12 s) groups (Table 5 and Table 6). While fall history was not recorded in the present analysis, the co-occurrence of greater gait variability, spending more time in the stance phase of the gait, and taking more time for each step might be mechanisms through which these individuals experience falls. Training both gait quality and multidirectional stepping abilities may be indicated for fall risk reduction in this group. Future work should longitudinally track fall rates and changes in the aforementioned gait performance measures to evaluate this association. Additionally, future work should investigate other characteristics of the slow FSST group, like body composition, coordination measures, and health history, to identify whether there are other markers which put this group at increased risk of mobility disability. Previous work in adults with interstitial lung disease found physiological functioning during the third minute of the 6MWT most strongly discriminated between physical functioning subgroups [14]. The present analysis did not find any meaningful interaction of time with FSST functional subgroups on gait performance measures (Table 5). In fact, visual inspection of gait performance between the FSST groups during the 6MWT reveals gait performance was most similar between groups in the third minute of the test in many cases, with a trend towards greater differences later in the test (Figure A2). Future work should evaluate fatigued gait performance to better distinguish those at greatest fall risk from their peers. This will guide whether training gait quality in a fatigued state might be a more effective intervention for those at greatest fall risk.

## 5. Conclusions

Among aging adults, demonstrating greater changes in gait performance throughout the 6MWT is associated with having lower self-reported and objectively measured physical functioning. Established FSST cut points are useful for discriminating those with poorer gait performance during the 6MWT from their peers. Future clinical assessments of the 6MWT should consider adding a single accelerometer to the lower leg to capture more sensitive measures of gait performance missed by the standard administration of the test. Fatigued gait quality may be an important clinical indicator of fall and mobility disability development risk which should be explored in future research.

## Figures and Tables

**Table 1:** Participant demographics (N = 21).

Age (years)		62.2 ± 6.4
BMI (kg/m^2^)		34.0 ± 6.4
Sex, male (n, %)		12, 57.1%
Assistive device (n, %)	None	17, 81.0%
	Cane	3, 14.3%
	Rollator	1, 4.8%
Race (n, %)	Black or African American	14, 66.7%
	White or Caucasian	7, 33.3%

**Table 2:** Gait performance measures summarized for each minute of the six-minute walk test. No significant effect of time was observed.

Gait Domain	Gait Performance Measure	Six-Minute Walk Test—Minute						*p*-Value
		1	2	3	4 ^a^	5	6	
Pace	Swing time COV (%)	3.47 ± 1.65	3.71 ± 2.32	3.70 ± 2.17	3.82 ± 2.49	3.70 ± 2.06	3.99 ± 2.31	*0.986*
Postural control	Stance percentage (% of stride)	63.87 ± 2.16	64.32 ± 2.11	64.45 ± 1.94	64.55 ± 1.96	64.58 ± 2.18	64.29 ± 1.97	*0.893*
Rhythm	Stride time (s)	1.10 ± 0.16	1.13 ± 0.18	1.14 ± 0.18	1.16 ± 0.19	1.15 ± 0.20	1.14 ± 0.19	*0.963*
	Stance time (s)	0.73 ± 0.12	0.73 ± 0.14	0.74 ± 0.14	0.75 ± 0.14	0.74 ± 0.16	0.73 ± 0.14	*0.954*
Variability	Stride time COV (%)	3.07 ± 1.04	3.46 ± 2.00	3.26 ± 1.10	3.41 ± 2.05	3.61 ± 2.97	3.84 ± 3.18	*0.905*
	Stance time COV (%)	4.11 ± 1.40	4.81 ± 3.67	4.28 ± 1.47	4.75 ± 3.58	4.88 ± 4.43	5.13 ± 4.87	*0.932*

^a^ N = 20.

**Table 3:** Heat map of correlations between change in gait performance measures with physical performance and self-reported measures of mobility. Darker values indicate stronger associations.

	Gait Speed (m/s)	Four Square Step Test (s) ^˄^	SF36 Physical Function	GHS Physical	GHS Mental	Daily Sedentary Activity (%)	Daily MVPA (%)	6-min Walk Test (m)
Increase in swing time variability	−0.372	0.412	−0.197	−0.059	0.136	−0.453	−0.028	−0.462 *
Increase in stance percent	−0.291	0.321	−0.283	−0.183	−0.085	−0.331	0.135	−0.364
Increase in stride time	−0.213	0.160	−0.594 **	0.023	−0.360	−0.156	−0.036	−0.394
Increase in stance time	−0.172	0.115	−0.679 **	0.007	−0.379	−0.123	−0.115	−0.362
Increase in stride time variability	−0.590 **	0.614 **	−0.273	0.219	−0.056	−0.410	0.003	−0.429
Increase in stance time variability	−0.433	0.498 *	−0.375	0.188	−0.152	−0.282	−0.028	−0.358

^^^ N = 19; ** *p* < 0.01; * *p* < 0.05; 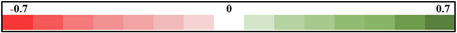.

**Table 4:** Heat map of frequency where maximum and minimum values for each gait parameter occurred. Darker shades of green indicate higher frequencies and lighter shades of yellow indicate fewer occurrences of extreme values in these minutes.

	Max Occurs in Minute:						Min Occurs in Minute:					
	1	2	3	4	5	6	1	2	3	4	5	6
Swing time COV	3	3	3	4	3	5	3	4	2	4	5	3
Stance percentage	5	3	4	2	5	2	4	5	2	6	0	4
Stride time	1	1	6	3	7	3	13	4	1	0	1	2
Stance time	1	1	5	4	5	5	15	3	0	1	0	2
Stride time COV	3	3	6	2	4	3	2	3	4	7	1	4
Stance time COV	5	3	4	1	5	3	5	3	1	3	3	6

**Table 5:** MANOVA results for a mixed model investigating the effects of time, FSST group, and their interaction on gait parameters during the six-minute walk test.

	Model	Intercept	Time	FSST Group	Time × FSST Group
Swing time variability	**R^2^ = 0.475** ***p* < 0.001**	**η_p_^2^ = 0.863** ***p* < 0.001**	η_p_^2^ = 0.015 *p* = 0.893	**η_p_^2^ = 0.463** ***p* < 0.001**	η_p_^2^ = 0.031 *p* = 0.967
Stance percentage	**R^2^ = 0.472** ***p* < 0.001**	**η_p_^2^ = 1.000** ***p* < 0.001**	η_p_^2^ = 0.040 *p* = 0.484	**η_p_^2^ = 0.457** ***p* < 0.001**	η_p_^2^=0.011 *p* = 1.000
Stance time	**R^2^ = 0.577** ***p* < 0.001**	**η_p_^2^ = 0.985** ***p* < 0.001**	η_p_^2^ = 0.028 *p* = 0.690	**η_p_^2^ = 0.570** ***p* < 0.001**	η_p_^2^ = 0.014 *p* = 0.999
Stride time	**R^2^ = 0.554** ***p* < 0.001**	**η_p_^2^ = 0.988** ***p* < 0.001**	η_p_^2^ = 0.021 *p* = 0.801	**η_p_^2^ = 0.547** ***p* < 0.001**	η_p_^2^ = 0.012 *p* = 0.999
Stance time variability	**R^2^ = 0.279** ***p* < 0.001**	**η_p_^2^ = 0.723** ***p* < 0.001**	η_p_^2^ = 0.036 *p* = 0.556	**η_p_^2^ = 0.214** ***p* < 0.001**	η_p_^2^ = 0.094 *p* = 0.358
Stride time variability	**R^2^ = 0.363** ***p =* 0.003**	**η_p_^2^ = 0.800** ***p* < 0.001**	η_p_^2^ = 0.046 *p* = 0.407	**η_p_^2^ = 0.305** ***p* < 0.001**	η_p_^2^ = 0.100 *p* = 0.306

**Bolded** indicates *p* < 0.05.

**Table 6:** Mean gait performance measure values for each FSST group and post hoc testing results. The slow group was significantly different from the fast and medium groups on all gait performance measures.

	FSST Group		
	≤12 s (N = 4)	12.01–14.99 s (N = 11)	≥15 s (N = 4)
Swing time variability (%)	3.07 ^a^	3.13 ^a^	6.71
Stance percentage (%)	63.30 ^a^	63.73 ^a^	66.18
Stance time (s)	0.66 ^a^	0.69 ^a^	0.91
Stride time (s)	1.04 ^a^	1.08 ^a^	1.37
Stance time variability (%)	4.15 ^a^	3.65 ^a^	7.79
Stride time variability (%)	2.93 ^a^	2.89 ^a^	5.85

^a^ significantly different from ≥15 s group at *p* < 0.001.

## Data Availability

The data presented in this study are available on request from the corresponding author. The data are not publicly available due to confidentiality commitments rendered to both VAMHCS R&D Committee and VAMHCS IRB of Record, as mandated by Privacy and Information Security Officers at the Baltimore VA Medical Center facility.
